# Author Correction: Contracting CAG/CTG repeats using the CRISPR-Cas9 nickase

**DOI:** 10.1038/s41467-024-52719-2

**Published:** 2024-10-17

**Authors:** Cinzia Cinesi, Lorène Aeschbach, Bin Yang, Vincent Dion

**Affiliations:** Center for Integrative Genomics, University of Lausanne, 1015 Lausanne, Switzerland

Correction to: *Nature Communications* 10.1038/ncomms13272, published online 09 November 2016

The original version of the Supplementary Information associated with this Article contained an error in Supplementary Table 4 in which the contents of plasmid pPN10-gCTG were incorrectly stated as “pPN10 with (CTG)_6_ gRNA – PAM: CTG” instead of “pPN10 with (CTG)_6_C gRNA – PAM: TGC”. The HTML has been updated to include a corrected version of the Supplementary Information.

## Supplementary information

Revised Supplementary Information

