# Supplementary material for: Contracting CAG/CTG repeats using the CRISPR-Cas9 nickase
Source: Nat Commun. 2016 Nov 9;7:13272. doi: 10.1038/ncomms13272 (PMC5105158; doi:10.1038/ncomms13272)
Supplement: Supplementary Information — Supplementary Figures 1-5, Supplementary Tables 1-8 and Supplementary References. [file ncomms13272-s1.pdf]

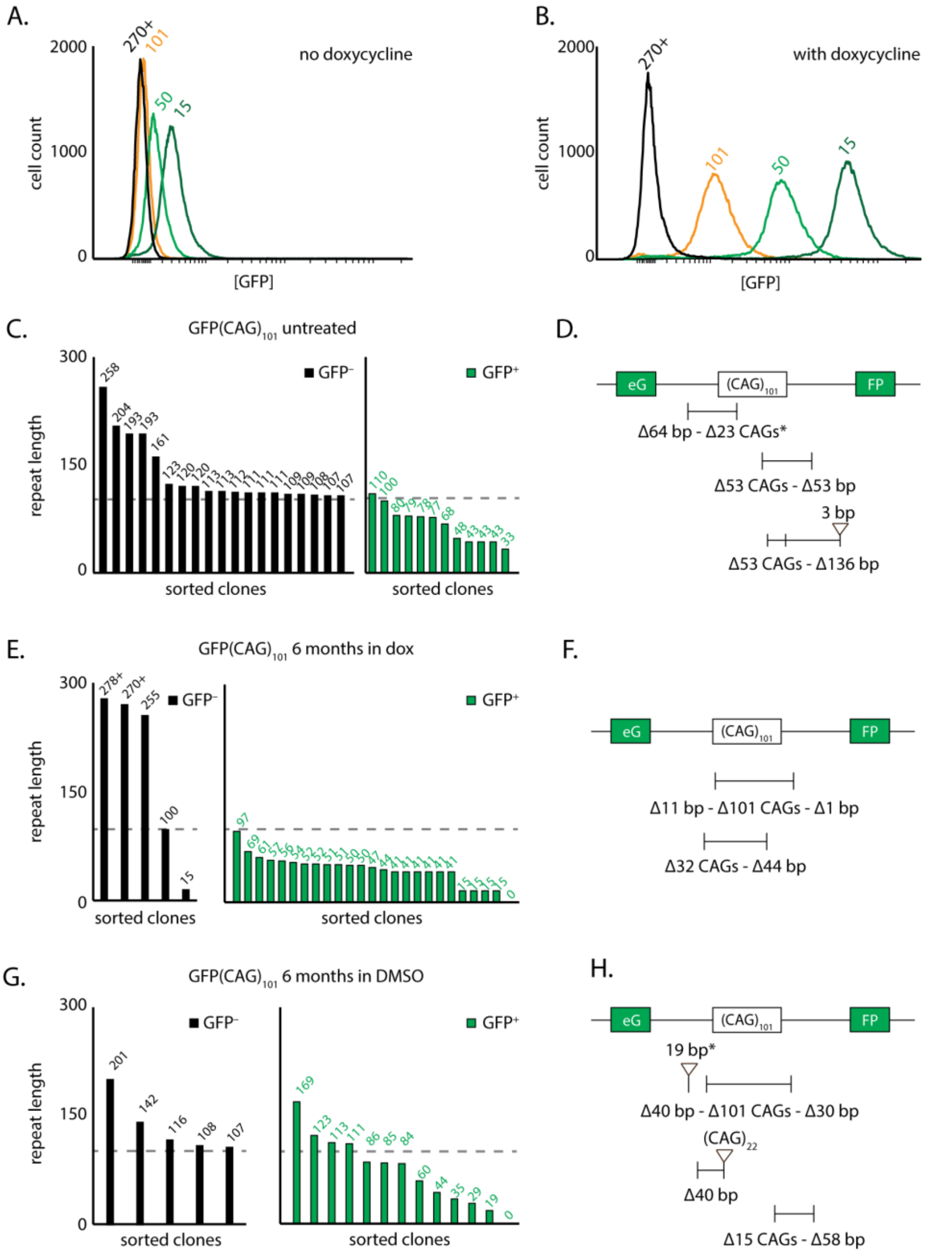

Supplementary Fig. 1: *Characterization of the GFP reporter assay and GFP$^-$ and GFP$^+$ cells isolated from GFP(CAG)$_{101}$.* A) Profile of GFP intensity in three cell lines isolated by FACS after six months of culturing compared to the starting population of GFP(CAG)$_{101}$. The repeat length in each clone is marked above the flow cytometry profiles. B) Same as A, but in the presence of 2µg/ml dox for 5 days. C) Repeat length for clones isolated from the GFP$^-$ and GFP$^+$ populations from GFP(CAG)$_{101}$ cells. The distributions of repeat lengths between GFP$^-$ and GFP$^+$ cells were significantly different (P=1x10^{-5}). D) Schematic representation of clones from C with mutations in the flanking sequences. *: Three different clones were isolated with the same deletion, two with 78 repeats, one with 77. E) Same as C, but with clones cultured in the presence of dox for 6 months. The distributions of repeat lengths between GFP$^-$ and GFP$^+$ cells were significantly different (P=0.025). F) Schematic representation of the deletions found after 6 months of culturing in the presence of dox. G) Same as E, except that the cells were exposed to DMSO. The distributions of repeat lengths between GFP$^-$ and GFP$^+$ cells were significantly different (P=0.035). H) Same as F, but for clones cultured in DMSO. *: The 19bp insertion is a direct repeat of the 19bp immediately found before the insertion.

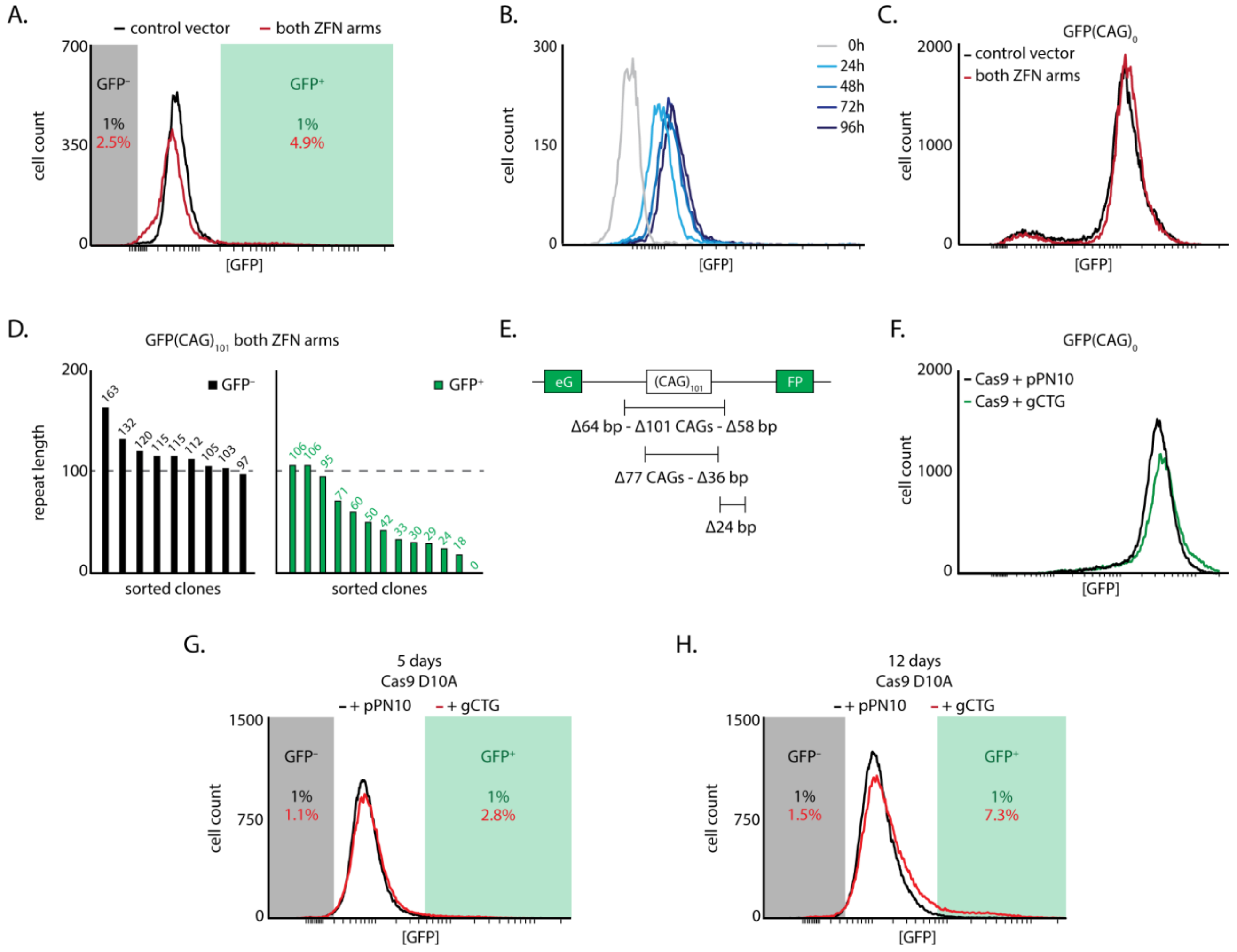

Supplementary Fig. 2: *Assay optimization, the effect of ZFN and Cas9 nuclease on GFP(CAG)$_0$ and analysis of GFP$^-$ and GFP$^+$ clones collected after ZFN treatment.* A) Example of data quantification. The GFP$^-$ and GFP$^+$ gates are set as the top or bottom 1% of the control population, in this case transfected with pcDNA3.1. The same gates are then used to determine the proportion of cells from the treated population that falls within these set gates have changed expression. B) Flow cytometry profile of cells treated with dox for an increasing amount of time. C) One of 10 flow cytometry experiments of GFP(CAG)$_0$ cells transfected with vectors expressing both ZFN arms or with a control vector (pcDNA3.1 Zeo). D) Repeat tract lengths in GFP$^-$ and GFP$^+$ clones after treatment of GFP(CAG)$_{101}$ cells with both ZFN arms. Dashed grey bars: repeat size in the starting population: 101 CAG repeats. The distributions of repeat lengths between GFP$^-$ and GFP$^+$ cells were significantly different (P=5x10^{-4}) E) Schematic representation of clones with deletions in the sequences surrounding the CAG repeat. F) One of two flow cytometry experiments comparing cells expressing the Cas9 nuclease and the gCTG or transfected with an empty gRNA vector (pPN10). G and H) Representative flow cytometry profiles showing that the number of GFP$^+$ cells increases after two more transfections over a total period of 12 days compared to our standard 5-day treatment.

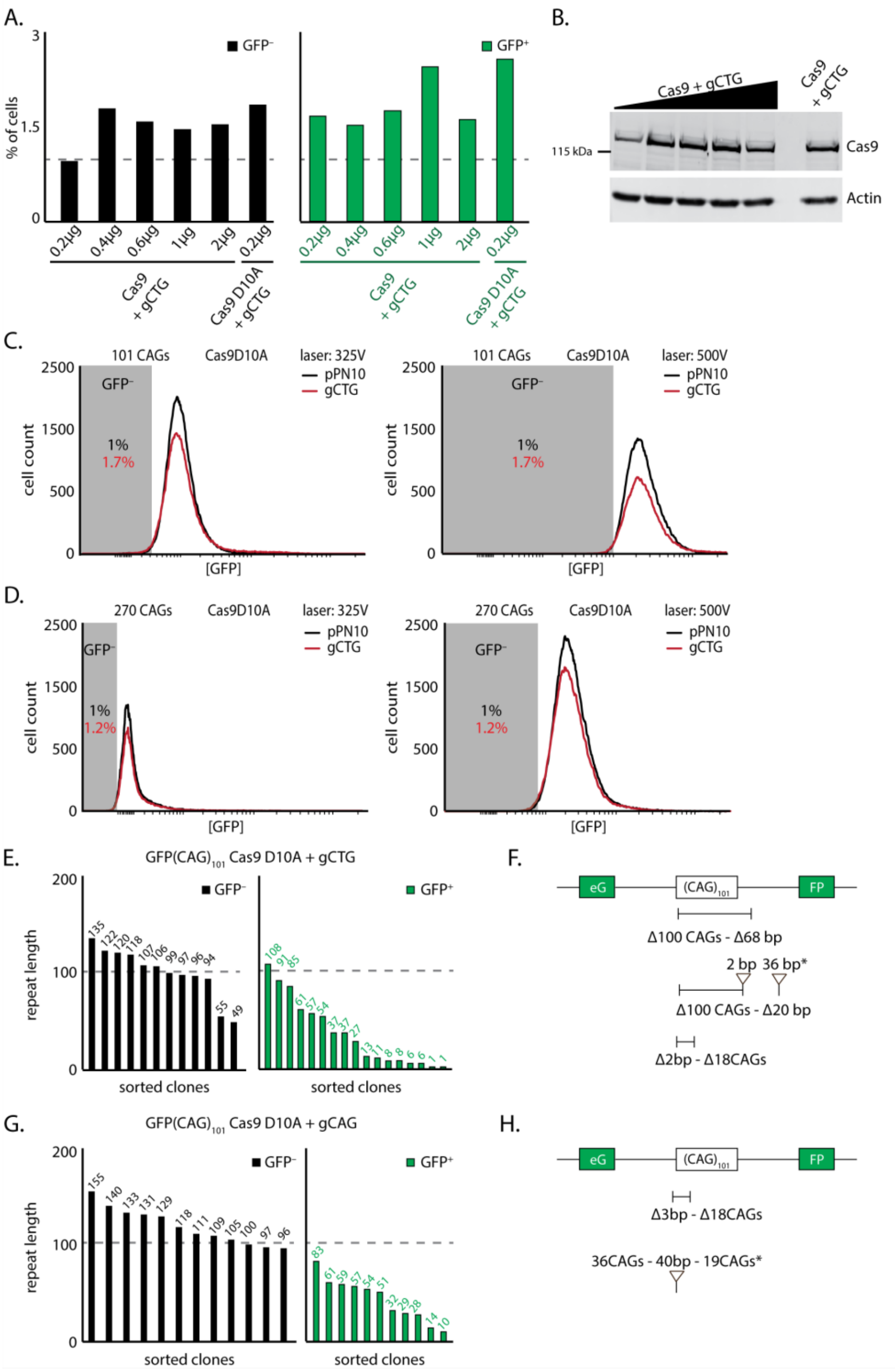

Supplementary Fig. 3: *Cas9 nickase induces repeat instability with a bias towards contractions.* A) Expression levels of the Cas9 nuclease and Cas9 nickase do not account for the different effects of these two enzymes on the number of GFP$^-$ and GFP$^+$ generated. Dashed line: dimmest (GFP$^-$) or brightest (GFP$^+$) 1% of the cells transfected with the indicated amount of the Cas9 nickase or nuclease vector together with the empty gRNA plasmid. B) Western of Cas9 levels for the experiment presented in (A). C) Flow cytometry data results from GFP(CAG)$_{101}$ cells transfected with the Cas9 nickase and with either pPN10 or gCTG-expressing vector showing that changing the laser intensity, and thus the apparent GFP expression, does not change the results of the quantifications. D) As in (C) but with GFP(CAG)$_{270}$. E) Size of repeat in clones isolated from GFP(CAG)$_{101}$ cells transfected with the gCTG and the Cas9-nickase expressing vectors. The distributions of repeat lengths between GFP$^-$ and GFP$^+$ cells were significantly different (P=2x10^{-4}). F) Schematic of the rearrangements from in 3 GFP$^+$ clones from (E). *: This clone contained a complex rearrangement with the 36bp insertion that includes a 10bp insertion followed by two direct repeats of 13bp corresponding to the last 13bp prior to the insertion. G) Same as in E, but with cells transfected with the Cas9 nickase together with gCAG. The distributions of repeat lengths between GFP$^-$ and GFP$^+$ cells were significantly different (P=1.5x10^{-6}). H) Schematic of the clones from (G) that had changes in the sequences flanking the repeat. *: This clone had a 19 CAG repeat expansions downstream of a duplication that included the 40bp immediately upstream of the repeat tract and 36 more CAGs.

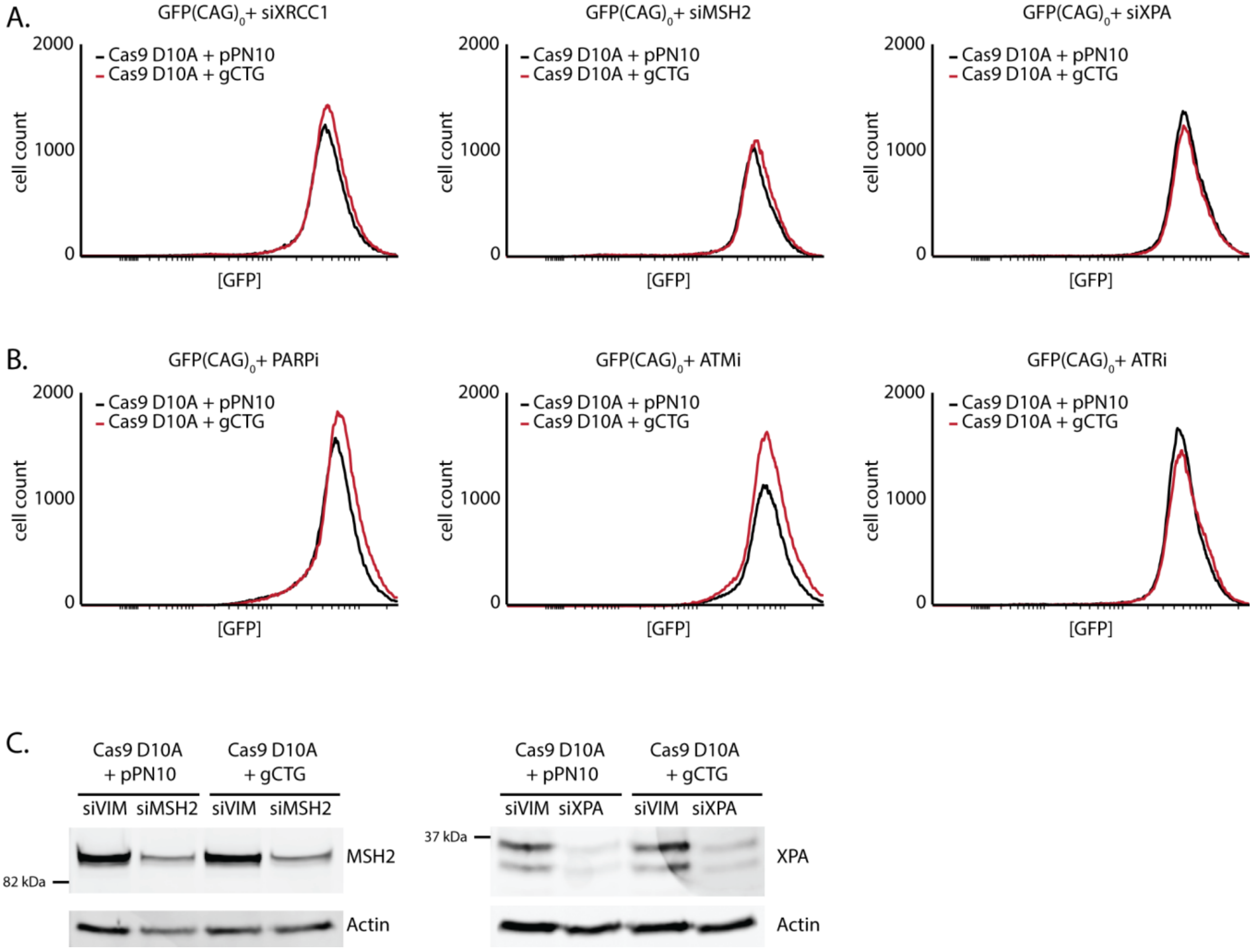

<u>Supplementary Fig. 4:</u> *Effect of siRNA and inhibitor treatments on GFP(CAG)$_0$ cells and knockdown efficiency*. A) Representative flow cytometry plots from siRNA knockdown experiments (*MSH2*: n=6; *XPA*: n=6; *XRCC1*: n=4). B) Representative flow cytometry results for inhibitor experiments (ATMi: n=5; ATRi: n=5; PARPi: n=4). C) Western blot showing knockdown efficiency by the *MSH2* and XPA siRNAs.

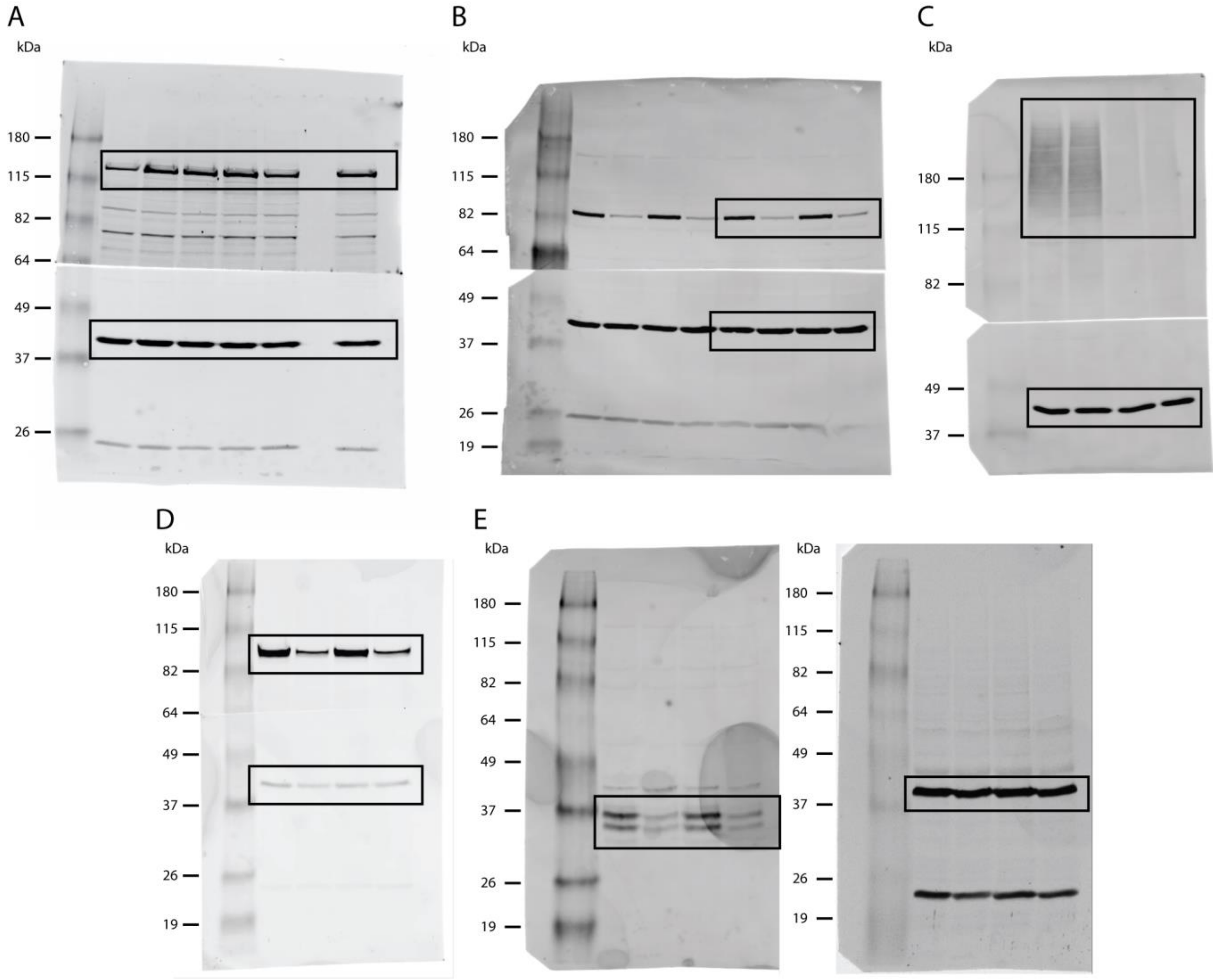

Supplementary Fig. 5: *Full size western blots.* A) Cas9 (top) and ACTIN (bottom) immunoblots from Supplementary Fig. 3B. B) XRCC1 (top) and ACTIN (bottom) blots from Fig. 3A. C) PARP (top) and ACTIN (bottom) western blots from Fig. 3B. D) MSH2 (top) and ACTIN (bottom) immunoblots from Fig. 4C. E) XPA (left) and ACTIN (right) immunoblots from Supplementary Fig. 4C. Boxes indicate the bands that were cropped.

Supplementary Tables

Treatment		Viability %*
pcDNA		76.6
ZFN 50		81.6
ZFN 51		79.8
ZFNs		75
Cas9 + pPN10		76.9
Cas9 + gDM1d		76.1
Cas9 + gCTG		85.4
Cas9 D10A + pPN10		77.3
Cas9 D10A + gDM1d		75.8
Cas9 D10A + gCTG	DMSO	81
	ATRi	82.6
	ATMi	77.2
	PARPi	75.9

Supplementary Table 1: Cell viability after transfection with the indicated plasmids and treatments.
*: derived from three experiments.

Locus	Sequence
AR	$(CAG)_{20-21}$-CAA GAG ACT AGC CCC AGG $(CAG)_5$
ATN1	CAG-CAA-CAG-CAA-$(CAG)_{15-16}$
ATXN1	$(CAG)_{12}$-CAT-CAG-CAT-$(CAG)_{11-12}$
DMPK	$(CTG)_5$
PPP2R2B	$(CAG)_{10}$
TBP	$(CAG)_3$-$(CAA)_3$-$(CAG)_9$-CAA-CAG-CAA-$(CAG)_{18-19}$-CAA-CAG
TCF4	$(CTG)_{14-17}$-$(CTC)_6$

Supplementary Table 2: Sequences of loci with CAG/CTG repeats in GFP$(CAG)_{101}$. Pure stretches are designated with parenthesis with the number of repeat as subscript. When two numbers are present, they refer to the number of repeats present on each allele.

Treatment	inhibitor	<2n	G1	S	G2	>4n
Cas9 D10A	DMSO	4.3 ± 0.5*	50.0 ± 1.1	18.8 ± 0.7	20.2 ± 1.4	6.2 ± 0.8
	ATMi	7.5 ± 0.8	34.9 ± 1.6	15.3 ± 1.7	37.2 ± 1.6	4.9 ± 1
	ATRi	2.0 ± 0.1	41.4 ± 1.4	20.9 ± 2.5	25.4 ± 2.2	10.3 ± 3
	PARPi	5.0 ± 0.4	40.7 ± 1.9	19.0 ± 2.2	30.0 ± 4.9	5.3 ± 1

Supplementary Table 3: Cell cycle analysis upon inhibitor treatment and Cas9 D10A transfection.
*: n=4 for each treatment. Average % of cells ± standard deviation.

Name	Content	Source
pcDNA3.1 Zeo	Empty vector	Life Technologies
pcDNA3.3-TOPO - Cas9_D10A	Cas9 D10A	[1] via Addgene
pcDNA3.3-TOPO hCas9	human Cas9	[1] via Addgene
pPN10	Empty gRNA	This study
pPN10-gCAG	pPN10 with $(CAG)_6$ gRNA – PAM: CAG	This study
pPN10-gCTG	pPN10 with $(CTG)_6$ gRNA – PAM: CTG	This study
pPN10-gDM1d	pPN10 with gRNA against the 3' UTR of the *DMPK* gene<br>target: TGCGAACCAACGATAGGTG PAM: GGG	This study
pZFN50	Single ZFN arm: 50	[2]
pZFN51	Single ZFN arm: 51	[2]

Supplementary Table 4: Plasmids using in this study. All plasmids created here are available upon request.

siRNA	Target	Sequence	Reference
siVIN-0001	*VIM*	GAAUGGUACAAAUCCAAGU	[3]
siVIN-0002	*MSH2*	UCUGCAGAGUGUUGUGCUU	[3]
siVIN-0003	*XPA*	GCUACUGGAGGCAUGGCUA	[3]
siVIN-0062	*XRCC1*	CAGUUUGUGAUCACAGCACAGGAAU	[4]

Supplementary Table 5: siRNAs used in this study.

Name inhibitor	Target	Concentration
Oliparib	PARP1/2	1 µM
KU60019	ATM	1 µM
VE-821	ATR	1 µM

Supplementary Table 6: Inhibitors used, their known target, and the concentration used in our experiments.

Primer	Locus	Sequence
oVIN-0437	Pem1 intron in the GFP cassette	TACCAGGACAGCAGTGGTCA
oVIN-0459	Pem1 intron in the GFP cassette	AAGAGCTTCCCTTTACACAACG
oVIN-0460	Pem1 intron in the GFP cassette	TCTGCAAATTCAGTGATGC
oVIN-1251	DMPK	GAGCGTGGGTCTCCGCCCAG
oVIN-1252	DMPK	CACTTTGCGAACCAACGATA
oVIN-1255	ATN1	ACTCAGCCTTCTCTCCCATC
oVIN-1256	ATN1	TGTAGGACACCTGGCTGTGA
oVIN-1257	AR	TAGGGCTGGGAAGGGTCTAC
oVIN-1258	AR	CTCTGGGACGCAACCTCTCT
oVIN-1259	ATXN1	TTCCAGTTCATTGGGTCCTC
oVIN-1260	ATXN1	GTGTGTGGGATCATCGTCTG
oVIN-1269	TBP	TTCTCCTTGCTTTCCACAGG
oVIN-1270	TBP	GGGGAGGGATACAGTGGAGT
oVIN-1273	PPP2R2B	GCAGCAAAGAGCAGCCGCAG
oVIN-1274	PPP2R2B	CTGGTCCCACGGGAGGGCGG

Supplementary Table 7: Primers used here with the locus targeted.

Antibody	Species	Dilution	Source	Reference
Anti-Actin	Rabbit	1:2000	Sigma-Aldrich	A2066-.2ML
Anti-CRISPR-Cas9	Rabbit	1:1000	Abcam	ab204448
Anti-MSH2 [3A2B8C]	Mouse	1:2000	Abcam	ab52266
Anti-PAR	Mouse	1:1000	Amsbio	4335-AMC-050
Anti-XPA [5F12]	Mouse	1:2000	Abnova	MAB6747
Anti-XRCC1 [33-2-5]	Mouse	1:1000	Abcam	ab1838

Supplementary Table 8: List of antibodies used, the dilution that we used for western blotting, the source and reference number.

Supplementary references

1. Mali, P. et al. RNA-guided human genome engineering via Cas9. *Science* **339**, 823-6 (2013).
2. Santillan, B.A., Moye, C., Mittelman, D. & Wilson, J.H. GFP-based fluorescence assay for CAG repeat instability in cultured human cells. *PLoS One* **9**, e113952 (2014).
3. Lin, Y., Dion, V. & Wilson, J.H. Transcription promotes contraction of CAG repeat tracts in human cells. *Nat Struct Mol Biol* **13**, 179-80 (2006).
4. Hubert, L., Jr., Lin, Y., Dion, V. & Wilson, J.H. Topoisomerase 1 and single-strand break repair modulate transcription-induced CAG repeat contraction in human cells. *Mol Cell Biol* **31**, 3105-12 (2011).
